# Maternal gestational diabetes mellitus and the childhood asthma in offspring: a meta-analysis

**DOI:** 10.1186/s13052-023-01532-6

**Published:** 2023-10-15

**Authors:** Xufeng Huang, Zhengguo Huang, Jing Zhang, You Jiang

**Affiliations:** Department of Pediatrics, Fuyang First People’s Hospital, No. 429 Beihuan Road, Fuyang District, Hangzhou, 311400 China

**Keywords:** Gestational diabetes mellitus, Asthma, Offspring, Risk factor, Meta-analysis

## Abstract

**Background:**

Maternal diabetes might be related to a high risk of allergic disease in offspring. However, it remains unknown if maternal gestational diabetes mellitus (GDM) is also associated with a high incidence of childhood asthma in offspring. A systematic review and meta-analysis was performed to investigate the above association.

**Methods:**

Relevant observational studies were obtained by search of electronic databases including Medline, Embase, Cochrane Library, and Web of Science. A randomized-effects model was selected to pool the data by incorporating the influence of potential heterogeneity. The Newcastle-Ottawa Scale was used for study quality evaluation. Subgroup analyses were performed to evaluate the potential influences of study characteristics on the outcome.

**Results:**

Ten datasets from seven moderate to high quality cohort studies, involving 523,047 mother-child pairs were included in the meta-analysis. Overall, maternal GDM was associated with a higher risk of childhood asthma in offspring (risk ratio [RR]: 1.22, 95% confidence interval [CI]: 1.07 to 1.39, p = 0.003; I^2^ = 30%). Subgroup analyses showed that the association was not significantly affected by study design, validation methods for GDM, or diagnostic strategy for asthma (p for subgroup analyses all > 0.05). The association between maternal GDM and asthma in offspring was more remarkable after adjusting maternal body mass index in early pregnancy (RR: 1.50 versus 1.06, p < 0.001), but significantly weakened after adjusting hypertensive disorders during pregnancy (RR: 1.08 versus 1.50, p = 0.001).

**Conclusions:**

Maternal GDM may be associated with an increased incidence of childhood asthma in offspring.

## Introduction

Asthma is a chronic and allergic disease which usually starts in childhood. Children with asthma experience respiratory symptoms such as wheezing, coughing, shortness of breath, chest tightness, as well as variable airflow limitation [1, 2]. Accumulating evidence suggests that prevalence of childhood asthma, as well as that of allergic diseases, has risen dramatically from the middle of the 20th century in developed countries, probably due to the changes of environmental exposures and lifestyles [3, 4]. Although children with asthma could be better controlled with standard pharmacological interventions, acute asthma attack may still happen as induced by factors such as respiratory infection. In fact, acute asthma attack has become one of the most common reasons for emergency department visit or hospitalization in children [5, 6]. Accordingly, it is important to identify risk factors for asthma pathogenesis. It has been suggested that a variety of maternal factors may be related to asthma pathogenesis in offspring, such as maternal smoking during pregnancy [7], pre-pregnancy maternal obesity [8], gestational weight gain [9], and gestational hypertensive disorders [10] etc. Besides, it has also been suggested that maternal diabetes may be a risk factor of allergic disease in offspring [11]. However, the potential association between maternal gestational diabetes mellitus (GDM) and the risk of asthma in offspring remains not fully understood [12, 13]. Therefore, in this study, we performed a systematic review and meta-analysis to investigate if maternal GDM is a risk factor of childhood asthma in offspring.

## Methods

We followed the Preferred Reporting Items for Systematic Reviews and Meta-Analyses (PRISMA 2020) [14, 15] and Meta-analysis Of Observational Studies in Epidemiology (MOOSE) [16] guidelines in the conducting and reporting of the meta-analysis.

### Selection of eligible studies

The PICOS criteria were used for study inclusion.

(1) P (Participants): Pregnant women;

(2) I (Intervention/exposure): With GDM during pregnancy;

(3) C (Control/comparator): No GDM during pregnancy;

(4) O (Outcome): Incidence of asthma in offspring;

(5) S (Study design): Observational studies, including cross-sectional studies, case-control studies, or cohort studies;

The diagnostic strategies for GDM and asthma were consistent with the methods applied in the original articles. Only studies published as full-length articles were included. Grey literatures, such as abstracts and unpublished data were excluded because these studies were typically not peer-reviewed, and inclusion of these studies may affect the reliability of the meta-analysis results. Reviews, editorials, meta-analyses, preclinical studies, studies that did not evaluate GDM during pregnancy, or studies that did not report the incidence of asthma in offspring were excluded. For studies with overlapped patients, the one with the largest sample size was included for the subsequent meta-analysis.

### Search of electronic databases

We identified relevant studies by a systematic search of Medline, Embase, Cochrane Library, and Web of Science electronic databases using the following search strategy: (“gestational diabetes” OR “GDM” OR (“gestational” OR “pregnancy” OR “pregnant”) AND (“diabetes” OR “diabetic” OR “hyperglycemia”)) AND (“asthma” OR “wheeze” OR “wheezing” OR “pulmonary” OR “lung” OR “allergy” OR “allergic”) AND (“child” OR “children” OR “adolescent” OR “pediatric” OR “pediatric” OR “infant” OR “neonate” OR “newborn” OR “toddler”). The search was from the inception of the databases to the date of last search (February 26, 2023). Only clinical studies published in English were selected. According the aim of the meta-analysis, only original studies were included. However, we also performed a manual check-up for the reference lists of the related original and review articles for potential identification of non-included original studies.

### Study quality evaluation and data collection

The Newcastle–Ottawa Scale (NOS) [17] was used for study quality assessment, which included three domains such as defining of study groups, between-group comparability, and validation of the outcome. A total of nine criteria were incorporated for the NOS, and one point was given if a certain criterion was met by the individual study. This scale totally scored from 1 to 9 stars, with 9 stars indicating the highest study quality level. Two of the authors independently conducted electronic database search, extraction of study data, and assessment of study quality according to the inclusion criteria described above. If there were discrepancies, discussion with the corresponding author was indicated to resolve them. The extracted data included the following: [1] study information (authors, countries, publication year, and study design); [2] numbers of mother-child pairs included, maternal age at index birth, methods for validation of GDM, and numbers of women with GDM; [3] age of children at the diagnosis of asthma, sex of offspring, methods for validation of asthma in children, numbers of children who developed asthma; and [4] variables included in the multivariate regression analysis for the association between maternal GDM and asthma in offspring.

### Statistical methods

Risk ratios (RRs) and 95% confidence intervals (CIs) were selected as the general outcome variable for the relationship between maternal GDM and the incidence of asthma in offspring. For studies that reported odds ratio (OR), data were converted to relative risks (RRs) for the meta-analysis as previously reported [18] (RR = OR/([1 − pRef]+[pRef×OR]), where pRef is the prevalence of the outcome in the reference group (non-GDM group). Data of RRs and standard errors (SEs) were calculated from 95% CIs or P values, and an additional logarithmical transformation was performed to stabilize variance and normalize to the distribution [19]. The Cochrane Q test was used to evaluate the heterogeneity, and the I^2^ statistic was also estimated [20]. Heterogeneity was deemed to be significant if I^2^ > 50%. We used a randomized-effects model for data synthesis because this model has incorporated the potential between-study heterogeneity and could provide a more generalized result [19]. Sensitivity analyses by excluding one dataset at a time were used to evaluate the stability of the findings. Subgroup analysis was performed to evaluate the association between maternal GDM and asthma in offspring according to study design, methods for validation of GDM and asthma, and adjustment of maternal body mass index (BMI) in early pregnancy or gestational hypertensive disorders. The funnel plots were constructed and a visual inspection of the symmetry was conducted to reflect the publication bias. The Egger’s regression asymmetry test was further performed for the evaluation of potential publication bias [21]. We used the RevMan (Version 5.1; Cochrane Collaboration, Oxford, UK) and Stata (version 12.0; Stata Corporation, College Station, TX) software for the statistical analyses.

## Results

### Results of database search

**Fig. 1:**
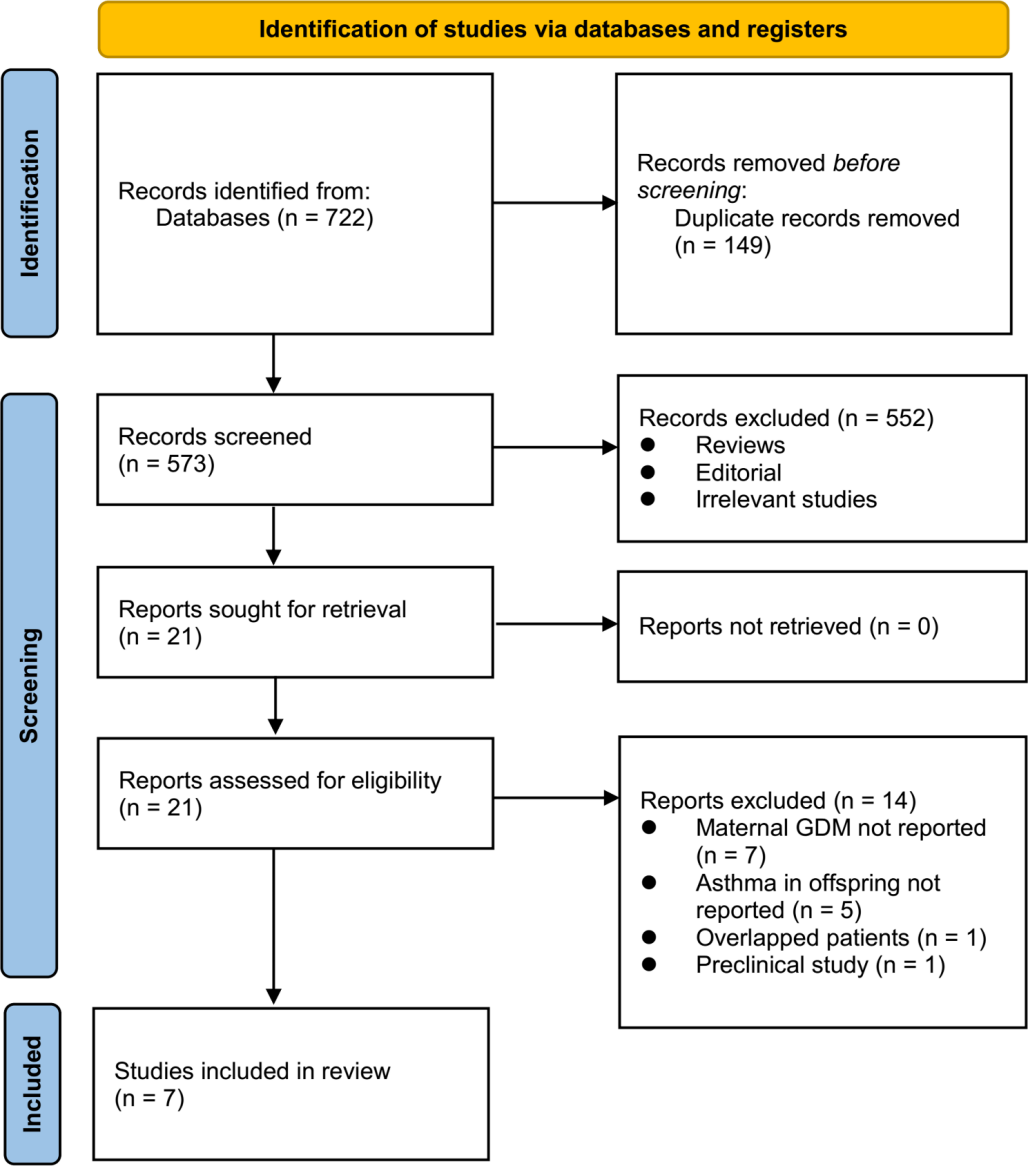
Flowchart of database search and study inclusion

The database search process is summarized in Fig. 1. Briefly, 722 articles were found in the initial literature search of the databases; after excluding the duplications, 573 studies remained. An additional 552 were excluded through screening of the titles and abstracts mainly because of the irrelevance to the meta-analysis. The remaining 21 studies underwent a full-text review, of which 14 were further excluded for the reasons listed in Fig. 1. Finally, seven observational studies [22–28] were included in the meta-analysis.

### Characteristics of the included studies

As shown in Table 1, seven cohort studies, which include two prospective cohorts [22, 23, 25, 27, 28] and five retrospective cohorts [22, 23, 25, 27, 28], were available for the meta-analysis. These studies were published between 2018 and 2023 and performed in Israel, the United States, Canada, China, and Greece, respectively. Overall, 523,047 mother-child pairs were included. The mean maternal age at index birth was 26.0 to 33.1 years. Medical records were reviewed for the validation of GDM in six studies [22–24, 26–28], while interview with questionnaires was used in the other study [25]. Accordingly, 46,286 (8.8%) of them had GDM in during the index pregnancy. As for the included children, they were evaluated for the diagnosis of asthma in 2 ~ 18 years, and the proportions of male were 49.8 ~ 54.5%. The validation of the diagnosis of asthma in offspring was achieved by review medical records in four studies [22–24, 28], by interview with their parents via questionnaires in two studies [25, 27], and by check the International Classification of Diseases codes of medical databases in another study [26]. A total of 44,683 (8.5%) of children developed asthma. Variables including demographic information of children and their mothers, ethnicity, maternal age at index birth, maternal smoking in pregnancy, and socioeconomic factors etc. were also adjusted to a different degree among the included studies. The NOS of the included studies were all 6 ~ 9 stars, suggesting moderate to good quality (Table 2).

**Table 1:** Summary of characteristics of the included studies

Study	Country	Design	Number of mothers included	Maternal age at index birth (years)	Methods for validation of GDM in mothers	Number of mothers with GDM	Number of children evaluated	Age of children at asthma diagnosis	Male offspring (%)	Methods for validation of asthma in children	No. of children with asthma	Variables adjusted/matched
Zamstein 2018	Israel	RC	216,197	28.4	Medical records	10,184	216,197	11.2 years, at least 4 years	51	Medical records	5,481	Maternal age, ethnicity, GA, hypertensive disorders of pregnancy, maternal smoking, and sex of offspring
Martinez 2020	USA	RC	97,554	30.2	Medical records	9836	97,554	Mean: 7.6 years, at least 5 years	51.4	Medical records	15,123	Birth year, maternal age, parity, education, household income, maternal race/ethnicity, history of comorbidity other than diabetes or asthma, history of maternal asthma, smoking during pregnancy, and sex of the child
Nasreen 2021	Canada	RC	19,933	29.2	Questionnaire	1178	19,933	6 ~ 15 years	50	Questionnaire	1,639	Maternal age at pregnancy, maternal smoking, maternal high blood pressure during pregnancy, multiple gestation, maternal educational attainment, annual household income, urban residency, and sex of the child
Adgent 2021	USA	PC	1,107	26	Medical records	62	1,107	3.7 ~ 6.5 years	49.8	Medical records	173	Maternal age, race, prenatal smoking, pre-pregnancy BMI, parity, asthma history, socioeconomic status, and sex of offspring
Dumas 2022	USA	PC	16,351	30.3	Medical records	769	16,351	3 ~ 5 years	52.1	ICD codes	2,306	Maternal age at delivery, asthma, maternal race/ethnicity, smoking status, insurance status at birth, mode of delivery, and early pregnancy BMI
Ma 2023	China	RC	166,772	27.8	Medical records	24,036	166,772	Mean: 8.6 years, 6 ~ 12 years	54.5	Questionnaire	19,722	Maternal age, educational level, BMI, maternal smoking during pregnancy, single child, family monthly income, children’s sex, and residential region
Papandreou 2023	Greece	RC	5,133	33.1	Medical records	221	5,133	2 ~ 5 years	NR	Medical records	239	Maternal age, pre-pregnancy overweight or obesity, educational and economic status, smoking habits, preterm birth, mode of delivery, breastfeeding practices, BMI, gestational weight gain and gestational hypertension

GDM, gestational diabetes mellitus; RC, retrospective cohort; PC, prospective cohort; NR, not reported; ICD, International Classification of Diseases; GA, gestational age; BMI, body mass index;

**Table 2:** Details of quality evaluation via the Newcastle-Ottawa Scale

	Representativeness of the exposed cohort	Selection of the non-exposed cohort	Ascertainment of exposure	Outcome not present at baseline	Control for maternal age	Control for other confounding factors	Assessment of outcome	Enough long follow-up duration	Adequacy of follow-up of cohorts	Total
Zamstein 2018	0	1	1	1	1	1	1	1	1	8
Martinez 2020	0	1	1	1	1	1	1	1	1	8
Nasreen 2021	0	1	0	1	1	1	0	1	1	6
Adgent 2021	1	1	1	1	1	1	1	1	1	9
Dumas 2022	1	1	1	1	1	1	0	1	1	8
Ma 2023	0	1	1	1	1	1	0	1	1	7
Papandreou 2023	0	1	1	1	1	1	1	1	1	8

### Meta-analysis results

**Fig. 2:**
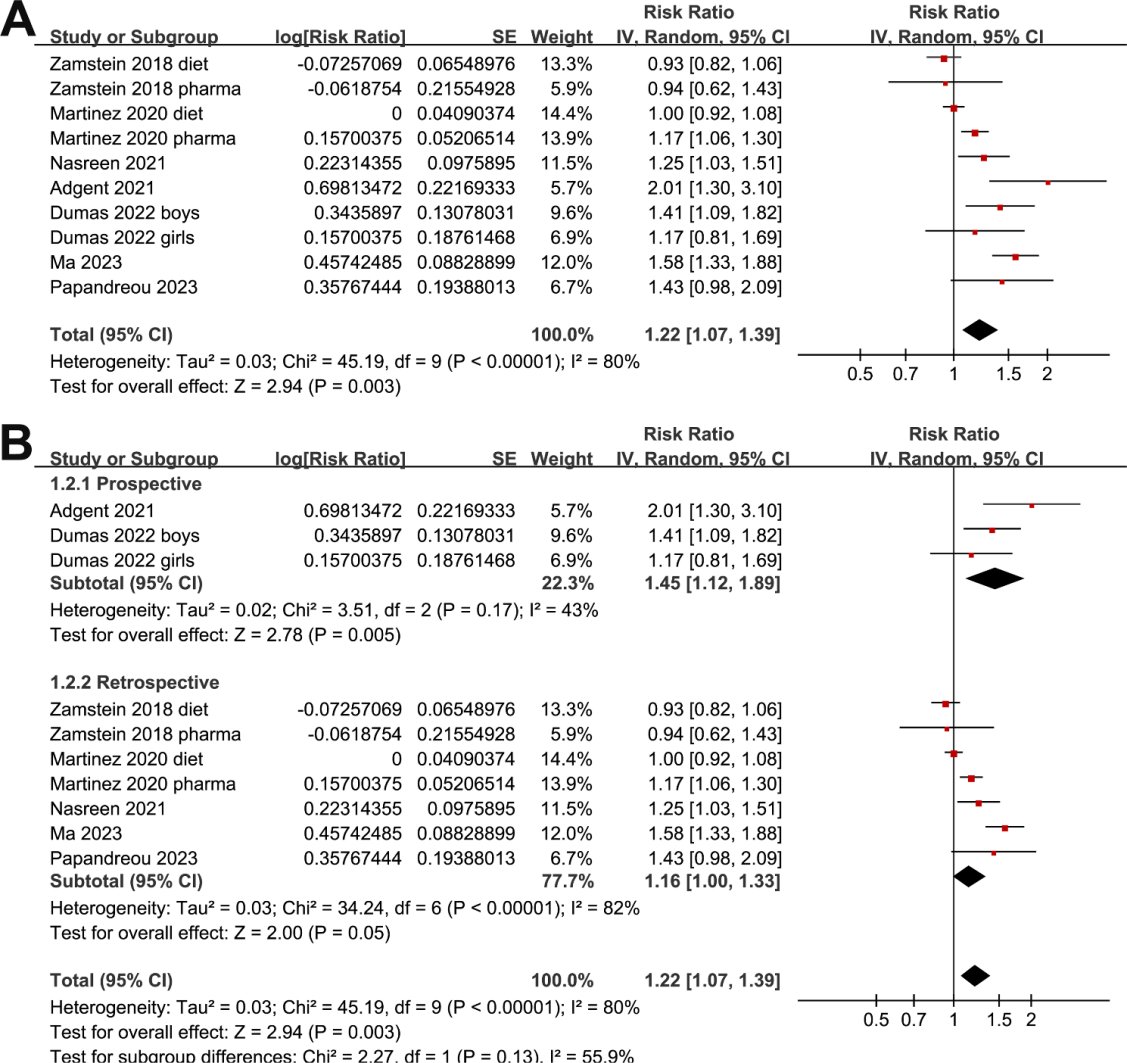
Forest plots for the meta-analysis of the association between maternal GDM and childhood asthma in offspring. **A**, forest plots for the overall meta-analysis; and **B**, forest plots for the subgroup analysis according to study design

**Fig. 3:**
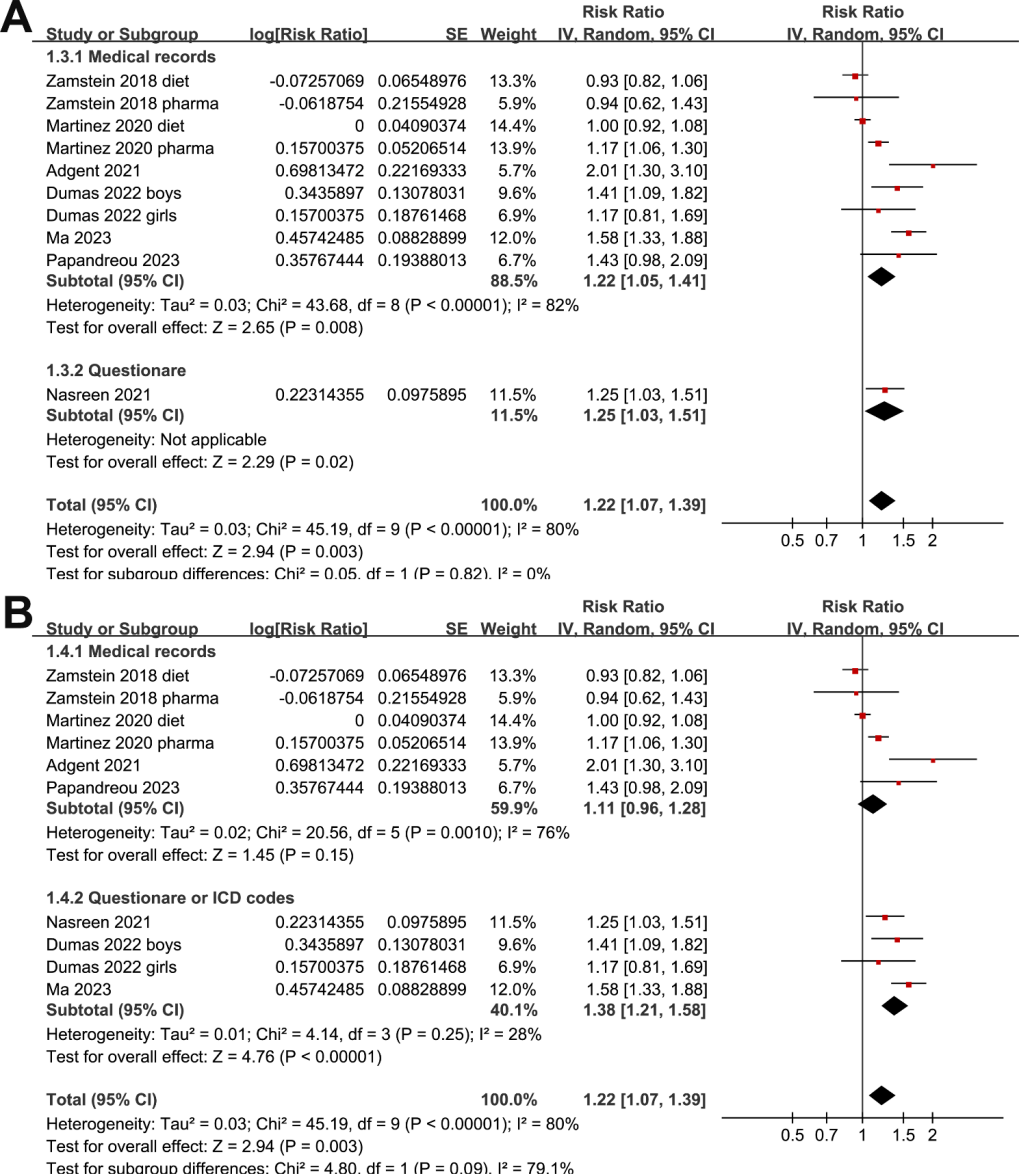
Forest plots for the subgroup analysis of the association between maternal GDM and childhood asthma in offspring. **A**, forest plots for the subgroup analysis according to validation methods for GDM; and **B**, forest plots for the subgroup analysis according to diagnostic strategy for asthma in offspring

**Fig. 4:**
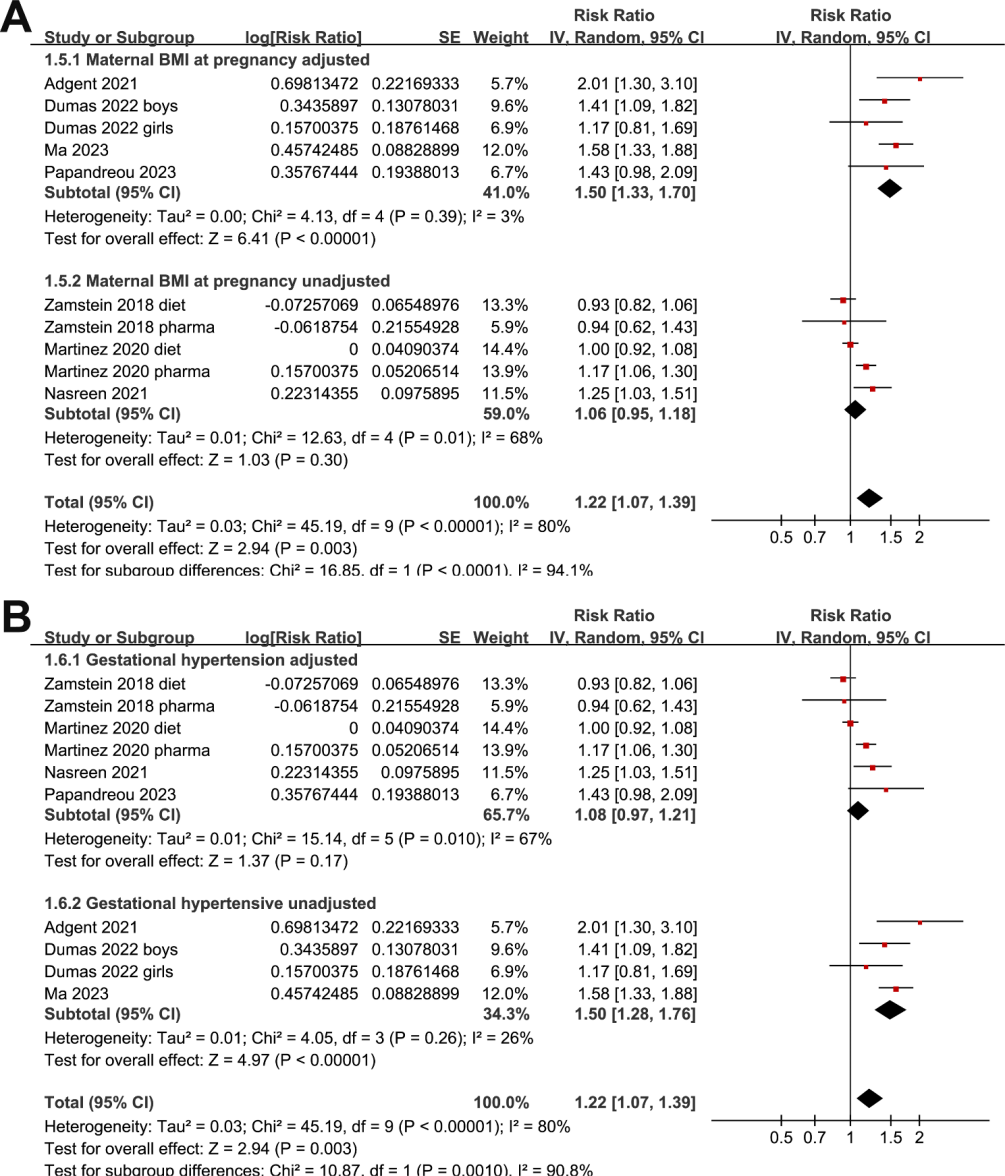
Forest plots for the subgroup analysis of the association between maternal GDM and childhood asthma in offspring. **A**, forest plots for the subgroup analysis according to adjustment of maternal BMI in early pregnancy; and **B**, **f**orest plots for the subgroup analysis according to adjustment of maternal hypertensive disorder in pregnancy

Since two studies reported outcomes in women with dietary-treated GDM and pharmacologically treated GDM [22, 23], and another one reported outcome according to the gender of the offspring [26], these datasets were included into the meta-analysis independently. Overall, ten datasets were included. Pooled results showed that maternal GDM was associated with a higher risk of childhood asthma in offspring (RR: 1.22, 95% CI: 1.07 to 1.39, p = 0.003; I^2^ = 30%; Fig. 2A). Sensitivity analyses by excluding one dataset at a time showed consistent result (RR: 1.16 to 1.27, p all < 0.05). Subgroup analyses showed that the association was not significantly affected by study design (Fig. 2B), validation methods for GDM (Fig. 3A), or diagnostic strategy for asthma (Fig. 3B, p for subgroup analyses all > 0.05). Interestingly, subgroup analysis showed that the association between maternal GDM and asthma in offspring was more remarkable in studies with adjustment of maternal BMI in early pregnancy as compared to those without adjustment maternal BMI (RR: 1.50 versus 1.06, p < 0.001; Fig. 4A). Moreover, the association was significantly weakened in studies with the adjustment of maternal hypertensive disorders during pregnancy as compared to those without adjustment of this factor (RR: 1.08 versus 1.50, p = 0.001; Fig. 4B).

### Publication bias

**Fig. 5:**
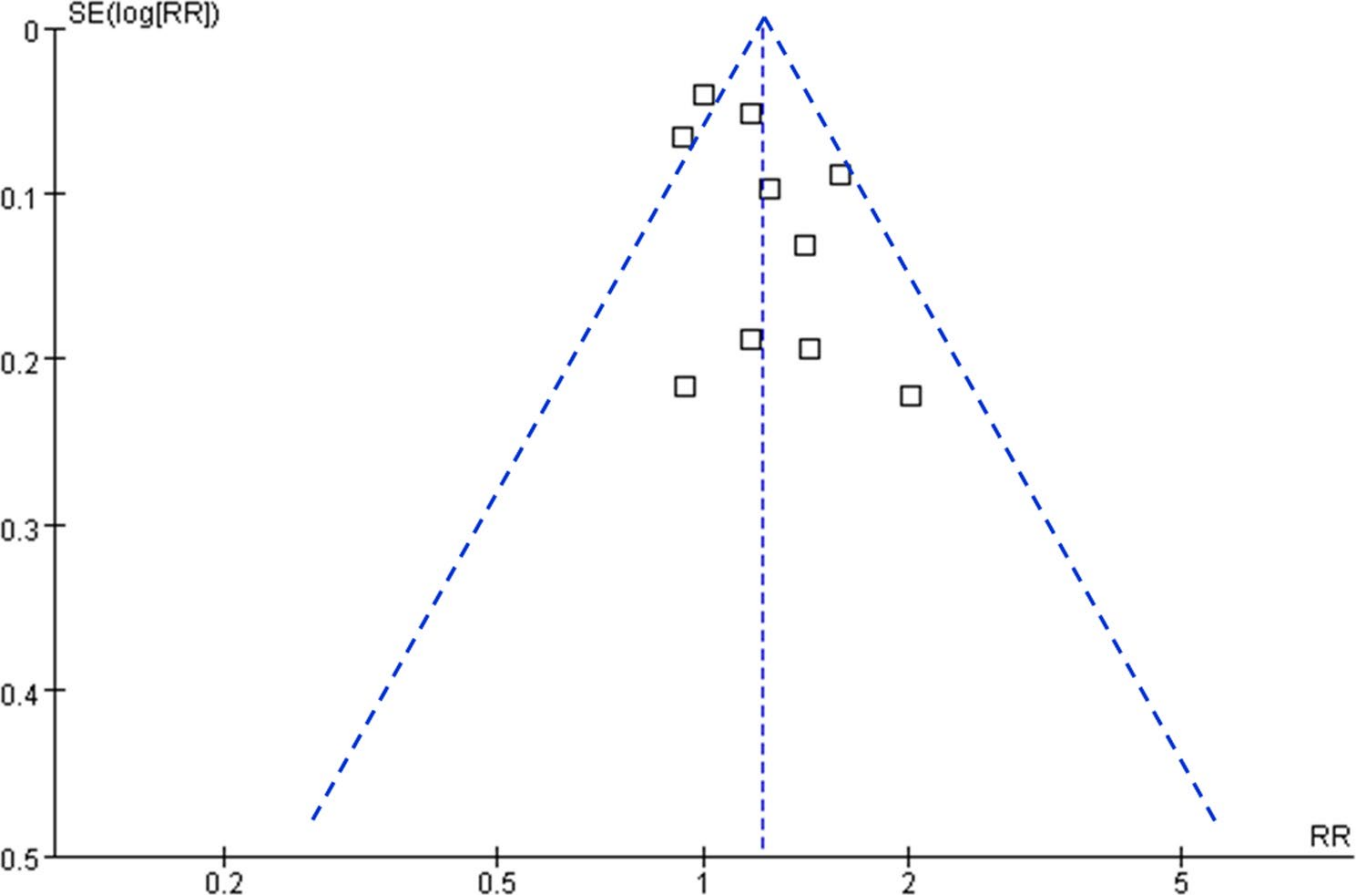
Funnel plots for the publication bias underlying the meta-analysis of the association between maternal GDM and childhood asthma in offspring

Figure 5 shows the funnel plots regarding the relationship maternal GDM and the risk of childhood asthma in offspring. Visual inspection found symmetry of the plots, which suggested a low risk of publication bias. Results of Egger’s regression tests also suggested low risk of publication bias (p = 0.68).

## Discussion

In this meta-analysis, we pooled the results of ten datasets from seven cohort studies and showed that maternal GDM may be associated with a higher risk of childhood asthma in offspring. The results were not significantly changed in sensitivity analysis by excluding one dataset at a time, suggesting the robustness of the finding. In addition, consistent results were obtained in subgroup analyses according to study design, validation methods for GDM, and diagnostic strategy for asthma. Moreover, the association between maternal GDM and the risk of childhood asthma in offspring was more remarkable in studies after adjustment of maternal BMI at early pregnancy, suggesting the association is independent of maternal obesity at pregnancy. Finally, the association was weakened in studies after adjustment of maternal gestational hypertensive disorders, suggesting the association between maternal GDM and the risk of childhood asthma in offspring may partly be confounded by the concurrent maternal gestational hypertensive disorders. Taken together, results of the meta-analysis suggest that maternal GDM may be a risk factor of childhood asthma in offspring.

To the best of our knowledge, few meta-analyses have been performed to investigate the potential influence of maternal GDM on the incidence of childhood asthma in offspring. As far as we know, only one previous meta-analysis evaluated the relationship between maternal diabetes in pregnancy and risk of allergic disease in offspring [11]. This meta-analysis included eight observational studies published before 2019 and showed that maternal diabetes mellitus may increase the risk of allergic diseases in their children, including asthma. However, studies evaluating maternal pregestational and GDM were both included in this meta-analysis, and for the outcome of childhood asthma, all of the included studies evaluated the influences of preexisting diabetes, rather than GDM [11]. Discrimination maternal pregestational (type 1 or type 2 diabetes) and GDM is important because they may have different pathophysiologic mechanisms [29] and different impacts on maternal and neonatal outcomes [30]. For example, one of the included studies showed that the risk of childhood asthma was predominately observed for exposure to maternal preexisting T2D, while was rater small for GDM [23].

Results of our meta-analysis further validated the hypothesis of the relationship between hyperglycemia in pregnancy and the risk of childhood asthma in offspring, by showing that maternal GDM may also be associated with childhood asthma. The methodological advantages of the meta-analysis may include the following. Frist, we extensively searched for relevant studies in four commonly used electronic databases, and seven up-to-date cohort studies were retrieved. In addition, only cohort studies were included, which could therefore provide a longitudinal association maternal GDM and childhood asthma in offspring. Moreover, multivariate analyses were used to estimate the association maternal GDM and childhood asthma in offspring in all the included studies, and potential confounding factors such as maternal age, smoking, and social economic factors etc. were adjusted. The results may therefore suggest a potentially independent association between maternal GDM and childhood asthma. At last, to further strengthen the robustness of the findings, multiple sensitivity and subgroup analyses also showed consistent results. Taken together, this meta-analysis confirmed that maternal GDM may be a risk factor of childhood asthma in offspring.

Subgroup analysis showed that the association between maternal GDM and childhood asthma was even stronger in studies after adjustment of maternal BMI in early pregnancy, suggesting the potential association was not confounded by maternal obesity in pregnancy. This is important because pre-pregnancy maternal obesity has been suggested as a possible risk factor for childhood asthma in offspring [9]. Interestingly, subgroup analysis also suggested that the association between maternal GDM and childhood asthma was weakened after adjustment of the prevalence maternal gestational hypertensive disorders. These findings may suggest that maternal GDM and gestational hypertensive disorders may share some similar mechanisms which may also be related to the pathogenesis of asthma in offspring, such as inflammatory response of interleukin balance [31] and hormonal changes involving the renin-angiotensin system [32].

This meta-analysis has indicated that GDM could potentially increase the risk of asthma in offspring. Consequently, interventions aimed at preventing GDM may also contribute to the prevention of asthma in the offspring. Various strategies, including lifestyle modifications, dietary supplementation, and pharmacological and non-pharmacological approaches, have been examined as potential means of preventing GDM. Among these interventions, adopting a healthy diet alone, combining a healthy diet with physical activity, supplementing with vitamin B complex, and implementing probiotic treatment have demonstrated promising outcomes in reducing GDM in high-risk women. However, further replication studies are necessary to validate these findings. The initial step in addressing this matter would involve identifying efficacious strategies for preventing GDM, with a particular focus on women who are predisposed to developing GDM. Subsequently, it would be imperative to assess the effectiveness of these measures in mitigating adverse outcomes in offspring, such as asthma.

Currently, the mechanisms underlying the association between GDM and childhood asthma in offspring remain to be elucidated. A recent preclinical study in a murine model of diet-induced GDM showed that female offspring exposed to GDM displayed increased methacholine reactivity, elevated proinflammatory cytokines in lung lavage, and an elevated abundance of matrix metalloproteinases in their airway, which all subsequently expose them to an increased risk of inflammatory lung conditions, such as asthma [33]. However, these changes were not observed in male offspring exposed to GDM [33]. Although studies are warranted to evaluate the underlying mechanisms and validate the potential offspring gender-specific relationship between exposure to maternal GDM and childhood asthma, this meta-analysis also has clinical implications. Again, these findings support the fetal origin hypothesis of the pathogenesis of asthma. On the other hand, considering that maternal GDM is a common risk factor for multiple poor outcomes in offspring, early monitoring and interventions should be offered to high-risk children of mothers with GDM. In addition, it is also interesting to evaluate whether optimizing the glycemic control of maternal GDM could reduce the risk of asthma in offspring.

This study also has limitations. First, studies available for the meta-analysis are limited, and more prospective cohort studies are needed to validate the finding. At current stage, we are unable to determine if the number of participants included in this meta-analysis is sufficient. Our meta-analysis is based on cohort studies aiming to evaluate if GDM is a risk factor of childhood asthma in offspring, and no intervention was involved. According, trial sequential analysis is not suitable for this meta-analysis because it is usually used for meta-analysis evaluating intervention effect to weigh type I and II errors and to estimate when the effect is large enough to be unaffected by further studies [34]. In addition, in some of the included studies, GDM or asthma in offspring were validated via questionnaire or ICD codes, which may affect the accuracy of the finding. Moreover, although multivariate analysis was used in all of the included studies when the association between maternal GDM and childhood asthma in offspring was estimated, we could not exclude the possibility that there may be residual factors confounding the association, such as maternal vitamin D [35] and fish oil supplementation [36]. Finally, this meta-analysis was on the basis of observational studies. Accordingly, a causative relationship between maternal GDM and childhood asthma in offspring could not be derived based on this meta-analysis.

## Conclusion

To sum up, results of the meta-analysis indicate that maternal GDM may be associated with an increased incidence of childhood asthma in offspring. Studies are needed to validate these findings and elucidate the underlying mechanisms. Moreover, studies are needed to determine if optimizing the glycemic control of women with GDM could reduce the incidence of asthma in their offspring.

## Data Availability

All data generated or analyzed during this study are included in this published manuscript.

## Abbreviations

BMI: Body mass index;
CIs: Confidence intervals;
GDM: Gestational diabetes mellitus;
NOS: Newcastle–Ottawa Scale;
PRISMA: Preferred Reporting Items for Systematic Reviews and Meta-Analyses;
RRs: Risk ratios;
SE: Standard errors;
